# Age and cohort rise in diabetes prevalence among older Australian women: Case ascertainment using survey and healthcare administrative data

**DOI:** 10.1371/journal.pone.0234812

**Published:** 2020-06-18

**Authors:** Befikadu L. Wubishet, Melissa L. Harris, Peta M. Forder, Julie E. Byles

**Affiliations:** 1 Research Centre for Generational Health and Ageing, Hunter Medical Research Institute, The University of Newcastle, Newcastle, New South Wales, Australia; 2 School of Pharmacy, Mekelle University, Mekelle, Tigray, Ethiopia; Nathan S Kline Institute, UNITED STATES

## Abstract

**Background:**

Due to the absence and or costliness of biological measures such as glycated haemoglobin, diabetes case ascertainment and prevalence studies are usually conducted using surveys or routine health service use databases. However, the use of each of these sources is associated with its limitations potentially impacting the quality of the case ascertainment and prevalence estimation. This study aimed at ascertaining diabetes cases and estimating prevalence among mid- and older-age women through simultaneous use of a longitudinal survey and multiple healthcare administrative data sources.

**Methods:**

Data were available for 12,432 and 13,714 women born in 1921–26 and 1946–51 from the Australian Longitudinal Study on Women’s Health (ALSWH). Diabetes was ascertained using the ALSWH survey, health service use, and cause of death data. Parsimonious multiple logistic regression analyses tested associations between sociodemographic and health variables and the presence of diabetes.

**Results:**

In both cohorts, two or more of the sources captured more than 80% of the women with diabetes. The point prevalence of diabetes increased from 8.4% when the mean age of the women were aged 73, to 22.0% of surviving women at age 90 in the 1921–26 cohort; and from 2.6% at age 48 to 15.8% at age 68 in the 1946–51 cohort. In the 1921–26 cohort, women who were obese (OR: 3.56; 95 CI: 3.04–4.17) and women who were sedentary (OR: 1.18; 95 CI: 1.09–1.40) were more likely to have diabetes compared to those who had a normal weight and engaged in a moderate level of physical activity. In the 1946–51 cohort, the odds of diabetes increased three times (OR: 2.99; 95 CI: 2.54–3.52) for overweight women and nine times (OR: 8.78; 95 CI: 7.46–10.33) for obese women compared to those who had normal weight.

**Conclusions:**

The simultaneous use of multiple data sources improved the validity of diabetes case ascertainment. Application of this methodology in future studies may have important benefits including estimation of disease burden, health service needs, and resource allocation with improved precision. Diabetes prevalence increased with age, was much higher in the 1946–51 cohort than in 1921–26 at similar ages, and was significantly associated with physical inactivity and obesity. Interventions to promote physical activity and a healthy weight are needed to prevent the rising prevalence of diabetes across successive generations.

## Introduction

Diabetes is one of four major non-communicable chronic diseases identified and prioritized by the World Health Organization due to its escalating prevalence and health and economic burden [1, 2]. Globally, diabetes prevalence has almost doubled (from 4.7% in 1980 to 8.5% in 2014) and the number of people living with diabetes has quadrupled (from 108 million in 1980 to 422 million in 2014) in less than four decades [3]. In 2017, 425 million of the world’s population (8.8%) aged 20–79 years were living with diabetes. Currently, this figure reached 463 million (9.3%) and is expected to grow to 700 million (9.9%) by 2045 [4, 5]. Type 2 diabetes, which is the focus of this study, accounts for up to 95% of the diabetes burden both globally and in Australia [6, 7].

In Australia, diabetes has been identified as a “national public health priority”. Its prevalence is estimated to be between 5.1% and 7.4%, affecting more than 1.2 million adults [8]. Mainly due to common complications of the disease, patients with diabetes usually have a lower quality of life, incur higher health care costs (at both a patient and health system level), and are at increased risk of premature mortality [9–11]. Up to 68% of Australians with diabetes develop serious complications such as cardiovascular disease, chronic kidney disease, and diabetic foot disease [12]. Diabetic foot disease is responsible for 27,600 public hospital admissions and 4400 lower extremity amputations and 1700 deaths costing the Australian health care system A$1.6 billion every year [13]. Diabetes also contributes to 10% of total deaths in Australia [12]. These figures imply that diabetes is a huge health and economic burden. Therefore, a reliable estimate of diabetes prevalence trends and identification of major determinants of observed trends is of high public health significance and may assist with the prioritization and monitoring of prevention efforts and optimal allocation of healthcare resources.

In estimating disease prevalence, it is important to pay attention to the case ascertainment method employed due to its impact on the precision of the prevalence estimation. For diabetes, obtaining biological measures such as measurement of haemoglobin A1c or oral glucose tolerance test (OGTT) for research purposes is expensive and usually not feasible. Therefore researchers often use other case ascertainment techniques such as surveys and health care administrative data sources (e.g. records of medication and other health service use or hospital use) [14]. However, there are a number of limitations to these data which may contribute to unreliable case ascertainment and biased prevalence estimation [15–17].

There is a possibility for recall bias in surveys especially when the recall period is long [18]. Many patients with diabetes may not be using diabetes-specific health services or prescribed diabetes medications. For instance, about a third of people with type 2 diabetes in Australia [19] and Canada [20] reported being treated with diet and lifestyle modification alone. Underreporting of diabetes in hospital admission data has been as high as 60% [21]. Moreover, most health care administrative databases have limitations such as partial coverage of the population and types of health services as well as missing records and errors in recording [22]. Therefore, there is potential to miss or misclassify some cases if using any single data source for case ascertainment and prevalence estimation [23]. Consequently, a better approach to case ascertainment is to use more than one data source [24]. In this study, we aimed to ascertain diabetes cases and estimate prevalence using longitudinal surveys and health care administrative data sources among women born in 1921–26 and 1946–51.

## Methods and materials

### Study design and data sources

This study involved data from the 1921–26 and 1946–51 cohorts of the Australian Longitudinal Study on Women’s Health (ALSWH). These two cohorts were recruited in 1996 using the Medicare database as a sampling frame and were aged 70–75 and 45–50, respectively, at the time of recruitment. Since 1996, women in both cohorts have been surveyed on a three-yearly rolling schedule, and the 1921–26 cohort women have completed six monthly surveys since 2011. The ALSWH survey data have been linked to multiple health care administrative databases including hospital admission, medication use, other health services use, cancer registry and death data. Detailed information on the ALSWH and linked data sources have previously been reported [25, 26] and are available on the study website: www.alswh.org.au. For this study, four main data sources were used to identify diabetes cases: the ALSWH survey, Pharmaceutical Benefits Scheme (PBS), Medicare Benefits Schedule (MBS), and Admitted Patients’ Data Collection datasets. The cause of death data from the National Death Index database was used as a fifth confirmatory source of data where necessary.

### ALSWH survey dataset

From the ALSWH survey data, a woman was considered to have diabetes if she responded “yes” to the question “Have you ever been told by a doctor that you have: diabetes (high blood sugar)?” in the first survey; or “In the last three years, have you been diagnosed with or treated for: diabetes?” in any of the later surveys. This analysis included up to six surveys for 12,432 women in the 1921–26 cohort and up to eight surveys for 13,714 women in the 1946–51 cohort. In survey 2 and survey 3, women from the 1946–51 cohort were asked to differentiate between type 1 and type 2 diabetes. It should be noted that most (for instance >99%) of the women in the 1946–51 cohort, had type 2 diabetes. Therefore, this study mainly focused on type 2 diabetes even though there is a possibility that a few women with type 1 diabetes may have been included.

### Medicare Benefits Scheme (MBS) dataset

MBS is designed to ensure access to medical services for all Australian citizens, permanent residents, and visitors from countries having reciprocal health care agreement with Australia [27]. Diabetes cases were identified from the MBS using item numbers for diabetes-specific services included under three major headings: 1) Annual Diabetes Cycle of Care; 2) HbA_1C_ and fructosamine testing for diabetes monitoring, and other diabetes services including eye examination and health education for patients with diabetes; and 3) group allied health services for people with type 2 diabetes.

### Pharmaceutical Benefits Scheme (PBS) dataset

PBS is an arrangement through which the Australian Government subsidizes the cost of most prescription medicines for eligible residents and visitors [28]. Identification of cases from the PBS dataset was performed using the World Health Organization’s Anatomic and Therapeutic Chemical Classification (ATC) codes. The medications indicating diabetes were: 1) insulin and insulin analogs (ATC codes: A10A) and; 2) blood glucose-lowering drugs (ATC codes: A10B).

### Hospital admission dataset

The Admitted Patients’ Data Collection (APDC), also called the *hospital admission dataset*, contains comprehensive data on hospital episodes. These include dates of admission and separation, and primary and secondary diagnoses based on the International Statistical Classification of Diseases and Related Health Problems, Tenth Revision, Australian Modification (ICD-10-AM) codes, procedures performed and inpatient stay costs [29]. Diabetes specific ICD-10-AM codes (E10, E11, E12, E13, and E14) were used to identify women with diabetes.

### Cause of death dataset

The National Death Index is an Australian Government database that contains records of all deaths in Australia since 1980. The data include name, sex, date of birth, and death details including cause of death and associated ICD-9 or ICD-10 codes [30]. This dataset was used only as supplemental data to ascertain diabetes status for women who self-reported diabetes only once or who had only a single record of health service or medication use indicating the presence of diabetes.

### Diabetes case definition

After compiling the diabetes indicators from each of the four data sources for the period 1996 to 2016, the following algorithms were used to assess the corroboration of the sources and have a robust ascertainment of diabetes status. A woman was considered to have diabetes if she had any of the following: i) a diabetes indicator in the hospital dataset; ii) at least one diabetes indicator from any two or more of the four main data sources; iii) two or more different PBS records for diabetes-specific medications; iv) two or more different MBS records for diabetes-specific MBS services; v) only one record specific to diabetes in PBS or MBS dataset, but had diabetes as a cause of death, or vi) reported diabetes in the ALSWH survey two or more times or reported only once but with diabetes coded as a cause of death.

Women who reported diabetes only at one survey, or who had only one PBS/MBS diabetes indicator and who had no other indicator of diabetes, even in the cause of death dataset, were considered to have uncertain diabetes status. These women were excluded from the analysis.

### Explanatory variables

Sociodemographic variables measured at baseline (Survey 1) included the area of residence (major cities, inner regional and outer regional/remote/very remote), highest educational qualification (year 12 or below, trade certificate/diploma, and a university degree or above), marital status (partnered versus not partnered), body mass index (BMI) [underweight (BMI<18.5), normal weight (18.5≤BMI<25), overweight (25≤BMI<30) and obese (BMI≥30)], difficulty managing on income (easy, difficult), smoking status (never smoker, ex-smoker, and current smoker), and private hospital insurance (yes or no). The level of physical activity (nil/sedentary, low, medium, and high) was measured at Survey 2. Missing responses at Survey 1 were backfilled by responses from Survey 2 or Survey 3 where logical.

### Statistical analyses

Venn diagrams were used to present diabetes cases identified from different data source combinations. Chi-squared tests were used to compare baseline characteristics of the women with and without diabetes. The likelihood of having diabetes was adjusted for sociodemographic, behavioral, and health variables using multiple multiple logistic regression models. The backward selection method at p<0.05 identified the most significant factors in parsimonious models. All statistical analyses were performed in SAS 9.4 (SAS Institute, Cary, NC, USA).

### Ethical considerations

The ALSWH has ongoing ethical clearance from the Human Research Ethics Committee of Universities of Newcastle and Queensland (approval numbers H0760795 and 2004000224, respectively), and all participants signed informed consent on joining the study. Ethical approval for linkage of the ALSWH survey data is also covered by the Universities (approval numbers H20110371 and 2004000224, respectively) as well as the NSW Population and Health Services Research Ethics Committee and other equivalent Committee for Admitted Patients Collections. Linkage to the National Death Index database was approved by the Australian Institute of Health and Welfare Ethics Committee.

## Results

### Case ascertainment

**Fig 1:**
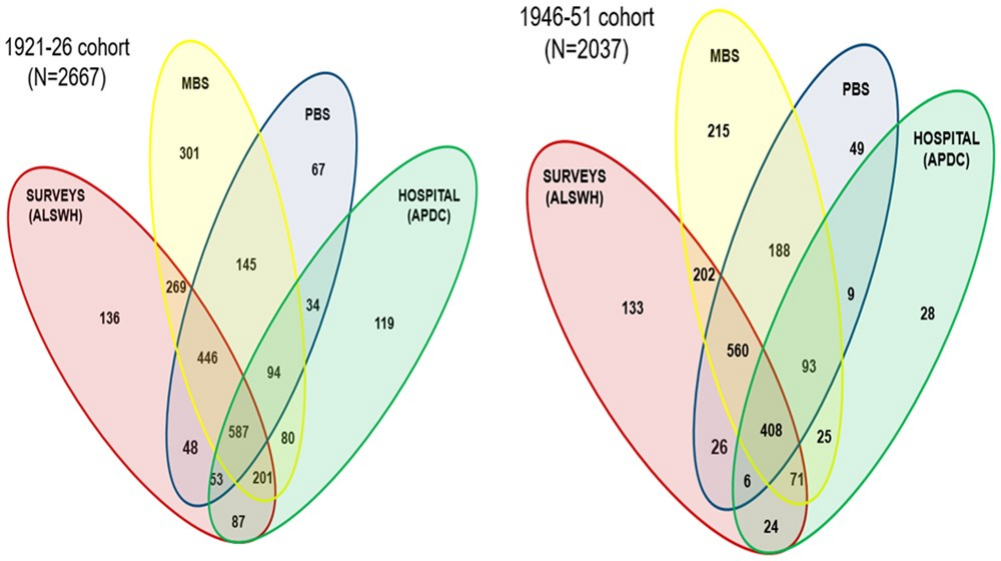
Number of women with diabetes identified from one or more data sources among women in the 1921–26 and 1946–51 cohorts for the period 1996–2016 [ALSWH: Australian Longitudinal Study on women’s Health; MBS: Medicare Benefits Scheme; PBS: Pharmaceutical Benefits Schedule; APDC: Admitted Patients’ Data Collection].

**Fig 2:**
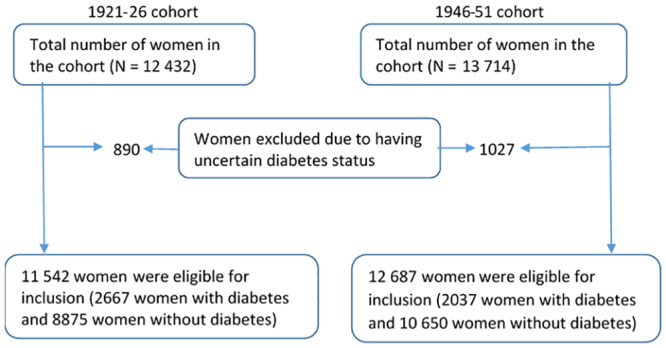
Derivation of the eligible populations from the 1921–26 and 1946–51 cohort women.

Diabetes cases identified from one or more of the data sources are presented in Fig 1. Of the 12432 and 13714 women in the 1921–26 and the 1946–51 cohorts, 890 and 1027, respectively, had uncertain diabetes status during the period 1 January 1996 to 31 December 2016. These women were excluded from the analysis (Fig 2). As shown in Fig 1 and S3 Table, the highest proportion of the diabetes cases were captured by the MBS data (79.6% and 86.5% in the 1921–26 and 1946–51 cohort women, respectively). The inclusion of survey and PBS datasets, respectively, captured the remaining 12.2% and 3.8% (in the 1921–26 cohort) and 9.3% and2.9% (in the 1921–26 cohort) of the diabetes cases.

#### 1921–26 cohort

**Fig 3:**
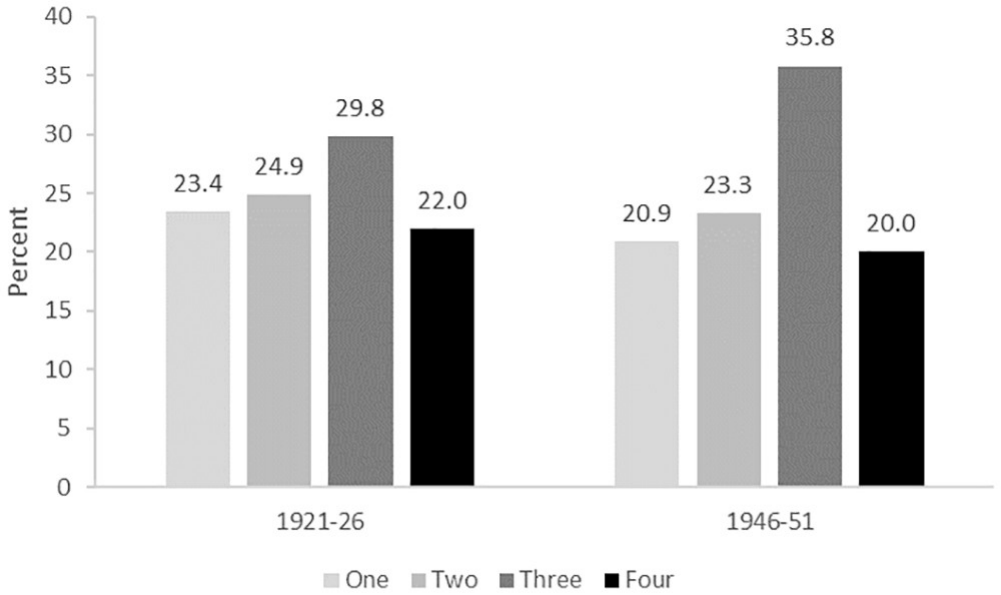
Percentage of diabetes cases identified according to the number of data sources for women in the 1921–26 and 1946–51 cohorts for the period 1996–2016.

After exclusion of uncertain cases, 2667 of the remaining 11542 women (i.e. 23.1%) were identified as having diabetes. Out of these women, 1691 (63.4%) were identified by both the ALSWH survey and at least one of the administrative data sources (MBS, PBS, or hospital) (Fig 3).

#### 1946–51 cohort

2037 of the remaining 12687 women in this cohort (16.1%) had diabetes. Diabetes was identified through survey data and at least one of the administrative sources for 1297 (63.7%) of the cases (Fig 3).

### Baseline characteristics

Table 1 presents a comparison of baseline characteristics for women who had and did not have diabetes (excluding women with uncertain diabetes). A significantly greater percentage of women with year 12 or below qualifications, those who were overweight or obese, not engaged in physical activity, and who had difficulty managing their income were identified with diabetes. Furthermore, a higher percentage of women without a partner (45.6% vs 42.8%) in the 1921–26 cohort; and those who were current smokers (22.0% vs 17.6%) and living in outer regional/remote/very remote areas (26.4% vs 25.1%) in the 1946–51 cohort had diabetes (p<0.0001).

**Table 1:** Univariate association between predictors and having diabetes among the 1921–1926 and 1946–51 cohort women.

	1921–26 cohort			1946–51 cohort		
Predictors	Have diabetes (n = 2667)	No diabetes (n = 8875)	P	Have diabetes (n = 2037)	No diabetes (n = 10650)	p
Highest level of education			<0.0001			<0.0001
Year 12 or below	2216 (88.1)	7073 (84.2)		1516 (75.7)	6876 (65.2)	
Trade certificate/diploma	224 (8.9)	998 (11.9)		303 (15.1)	2100 (19.9)	
University degree	75 (3.0)	328 (3.9)		184 (9.2)	1577 (14.9)	
Area of residence			0.11			0.44
Major cities	1090 (40.9)	3675 (41.4)		721 (35.4)	3879 (36.4)	
Inner regional	1012 (38.0)	3479 (39.2)		778 (38.2)	4094 (38.4)	
Outer regional/remote/very remote	565 (21.2)	1721 (19.4)		538 (26.4)	2676 (25.1)	
Marital status			0.0090			0.0575
Partnered*	1444 (54.4)	5066 (57.2)		1650 (81.4)	8805 (83.1)	
Not partnered	1212 (45.6)	3786 (42.8)		378 (18.6)	1791 (16.9)	
BMI classification			<0.0001			<0.0001
Normal weight (18.5≤BMI<25)	902 (35.4)	4618 (54.0)		413 (21.6)	5820 (56.6)	
Underweight (BMI<18.5)	35 (1.4)	339 (4.0)		11 (0.6)	207 (2.0)	
Overweight (25≤BMI<30)	979 (38.4)	2684 (31.4)		615 (32.1)	2888 (28.1)	
Obese (BMI≥30)	634 (24.9)	913 (10.7)		877 (45.8)	1377 (13.4)	
Level of physical activity			<0.0001			<0.0001
Moderate	305 (14.3)	1136 (16.0)		338 (21.3)	1961 (22.3)	
Nil/sedentary	870 (40.7)	2294 (32.2)		383 (24.1)	1493 (17.0)	
Low	609 (28.5)	2144 (30.1)		485 (30.6)	2707 (30.8)	
High	353 (16.5)	1546 (21.7)		381 (24.0)	2627 (29.9)	
Smoking status			<0.0001			<0.0001
Never smoked	1604 (64.8)	5134 (61.8)		982 (50.1)	5555 (53.8)	
Ex-smoker	728 (29.4)	2470 (29.7)		547 (27.9)	2946 (28.6)	
Current smoker	142 (5.7)	710 (8.5)		430 (22.0)	1817 (17.6)	
Difficulty to manage on income			0.0004			<0.0001
Easy	1883 (71.1)	6578 (74.5)		946 (46.7)	6175 (58.3)	
Difficult	767 (28.9)	2251 (25.5)		1078 (53.3)	4409 (41.7)	
Private hospital insurance			<0.0001			<0.0001
No	4754 (54.0)	1582 (59.8)		1178 (58.0)	5473 (51.5)	
Yes	4054 (46.0)	1062 (40.2)		854 (42.0)	5165 (48.6)	

*Married or in a de facto relationship, BMI: Body mass index

### Prevalence

**Fig 4:**
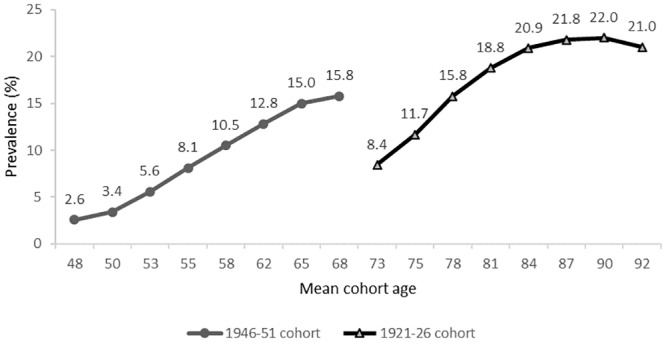
Comparison of age and cohort rise in diabetes prevalence among the 1921–26 and 1946–51 cohort women for the period 1996 to 2016.

As shown in Fig 4, the point prevalence of diabetes among the 1921–26 cohort women increased from 8.4% when the mean age of the women was 73 and reached 22.0% of surviving women when they were aged 90. In the 1946–51 cohort, the prevalence of diabetes increased from 2.6% when the women were aged 48 and reached 15.8% when they were aged 68. The prevalence in the 1946–51 cohort at age 58 was already higher than the prevalence in the 1921–26 cohort at age 73 (10.5% vs 8.4%, respectively).

### Predictors of having diabetes

As shown in Table 2, women who were overweight and obese at baseline had greater odds of having diabetes compared to those women with normal BMI, increasing nine-fold for women in the 1946–51 cohort who were obese compared to women with a normal weight. In both cohorts, there was about a 15% reduction in the odds of having diabetes among women with high levels of physical activity compared to women with moderate levels of physical activity. Being sedentary was associated with an 18% and 11% increase in the odds of having diabetes among the 1921–26 and 1946–51 cohort women, respectively. Furthermore, women in the 1946–51 cohort had a 55% increased likelihood of having diabetes if they were current smokers and 45% increased likelihood if they had difficulty managing their income.

**Table 2:** Multivariate association between predictors and having diabetes among the 1921–1926 and 1946–51 cohort women.

	1921–26 cohort				1946–51 cohort			
	Full model		Parsimonious model		Full model		Parsimonious model	
Predictors	Odds ratio (95% CI)	p	Odds ratio (95% CI)	p	Odds ratio (95% CI)	p	Odds ratio (95% CI)	P
Highest level of education								
Year 12 or below	1.00		1.00		1.00			
Trade certificate/diploma	0.76 (0.63–1.09)	0.14	0.74 (0.62–1.08)	0.23	0.79 (0.67–1.09)	0.16	0.79 (0.67–1.06)	0.59
University degree	0.75 (0.55–1.01)	0.10	0.73 (0.54–1.09)	0.29	0.69 (0.56–0.85)	0.0197	0.70 (0.57–0.86)	0.0205
Area of residence								
Major cities	1.00				1.00			
Inner regional	1.01 (0.89–1.15)	0.25			0.92 (0.79–1.07)	0.29		
Outer regional/remote/very remote	1.17 (1.00–1.37)	0.30			1.01 (0.85–1.20)	0.81		
Marital status								
Partnered*	1.00				1.00			
Not partnered	1.08 (0.96–1.22)	0.29			0.93 (0.78–1.11)	0.41		
BMI classification								
Normal weight (18.5≤BMI<25)	1.00		1.00		1.00		1.00	
Underweight (BMI<18.5)	0.63 (0.40–0.99)	<0.0001	0.63 (0.40–1.21)	0.07	1.03 (1.01–2.06)	0.002	1.04 (1.01–2.07)	0.25
Overweight (25≤BMI<30)	1.91 (1.68–2.18)	<0.0001	1.94 (1.71–2.21)	<0.0001	3.00 (2.55–3.54)	0.0055	2.99 (2.54–3.52)	0.0060
Obese (BMI≥30)	3.48 (2.96–4.08)	<0.0001	3.56 (3.04–4.17)	<0.0001	8.82 (7.49–10.39)	<0.0001	8.78 (7.46–10.33)	<0.0001
Level of physical activity								
Moderate	1.00		1.00		1.00		1.00	
Nil/sedentary	1.18 (1.01–1.40)	0.0013	1.18 (1.09–1.40)	0.0010	1.11 (1.09–1.34)	0.0139	1.11 (1.02–1.35)	0.0125
Low	1.03 (0.86–1.22)	0.76	1.02 (0.86–1.22)	0.79	0.89 (0.75–1.07)	0.18	0.89 (0.75–1.07)	0.18
High	0.84 (0.65–0.91)	0.0057	0.86 (0.71–0.93)	0.006	0.85 (0.71–0.94)	0.0316	0.85 (0.71–0.91)	0.02
Smoking status								
Never smoked	1.00				1.00		1.00	
Ex-smoker	1.00 (0.88–1.14)	0.30			1.11 (0.97–1.28)	0.014	1.12 (1.07–1.29)	0.14
Current smoker	0.84 (0.65–1.08)	0.21			1.52 (1.28–1.81)	<0.0001	1.55 (1.31–1.84)	<0.0001
Difficulty to manage on income								
Easy	1.00				1.00		1.00	
Difficult	1.08 (0.95–1.23)	0.26			1.42 (1.24–1.62)	<0.0001	1.45 (1.27–1.64)	<0.0001
Private hospital insurance								
No	1.00				1.00			
Yes	0.92 (0.81–1.03)	0.25			0.93 (0.81–0.96)	0.28		

*Married or in a de facto relationship, BMI: Body mass index

## Discussion

This study ascertained diabetes cases among Australian women born in 1921–26 and 1946–51 using longitudinal survey data and multiple health care administrative data sources including hospital admission, PBS, and MBS. We found a high level of concordance between survey and health care administrative data sources in identifying patients with diabetes. The study showed the added advantage of simultaneously using multiple data sources to improve the reliability and completeness of diabetes case ascertainment and precision of prevalence estimation even though some women with diabetes may have been falsely classified as uncertain. In the 1921–26 cohort women, diabetes prevalence increased from 8.4% at a mean age of 73 to 22.0% when they were aged 90. In the 1946–51 cohort women, there were relatively greater increments in diabetes prevalence at earlier ages. In both cohorts being overweight, being obese, and being sedentary were significantly associated with an increased likelihood of having diabetes, with the impact of these affecting at earlier ages for the 1946–51 cohort. These findings have important implications for the prevention and management of diabetes.

Compared to other research that employed a single survey or administrative database [31, 32], a more robust ascertainment of diabetes cases was achieved in this study through the simultaneous use of four main data sources. This has resulted in improved completeness and validity of the diabetes case ascertainment. For instance, if we were using only the MBS dataset, the dataset which captured the highest proportion of cases in both cohorts, we would have missed 20.4% (1921–26 cohort) and 13.5% (1946–51 cohort) of the cases. If this was done, it would underestimate the diabetes burden and also potentially the health service planning and resource allocation for the disease. The finding that approximately 20% of women with diabetes had diabetes indicators in all of the four data sources indicates the improvement in the validity of the case ascertainment. This proportion is high compared to a previous study that used similar data sources to ascertain dementia among women in the 1921–26 cohort, which found only 2–3% of the women being identified by four of the data sources [24]. This suggests the existence of better concordance between these data sources in identifying diabetes cases compared to other diseases such as dementia. Other studies that showed higher agreement between data sources for diabetes than for other conditions (eg. hypertension) suggest that this may be due to diabetes requiring ongoing self-management and regular health service use [33, 34].

This study also highlights the value of longitudinal, population-based data collection as part of routine health care service provision to be used in research. The case ascertainment methodology developed in this study can be applied in other settings and disease conditions resulting in enhanced reliability of case ascertainment and precision of prevalence estimation. Such precise disease prevalence estimates may have numerous practical benefits including correctly estimating disease burden, planning of needed health services, and resource allocation. This is particularly important to diseases where ‘gold standard’ methods of ascertainment are not feasible for widespread monitoring, but where disease indicators can be found in multiple health care administrative data sources.

Our study confirmed cohort differences and identified increasing agewise trends in diabetes prevalence previously hypothesized in cross-sectional studies [35–37]. The prevalence of diabetes continuously increased with age during the entire follow-up period among both cohorts of women. However, women in the 1946–51 cohort had prevalence levels that were similar to the prevalence observed among the 1921–26 cohort women when the former were aged 15–20 years younger. Even though adjustment for overweight/obesity narrowed the gap in the prevalence of diabetes in the two cohorts, the prevalence was still higher among the 1946–51 cohort women. Higher diabetes prevalence patterns at younger ages were also observed among the 1973–78 and 1989-95 cohorts of ALSWH [38]. This may mean that future cohorts of women are expected to have a higher prevalence of diabetes at much younger ages.

The odds of having diabetes were observed to be higher for women who were overweight or obese, those who had difficulty managing their available income, those who were current smokers, and women who had a lower level of education. Among the 1921–26 cohort women, being overweight and obese as well as having a sedentary lifestyle was associated with increased odds of having diabetes. These findings are in line with previous studies [39, 40]. However, what is worth noting is that especially for the 1946–51 cohort women, being obese had a highly pronounced (~9 times) association with having diabetes compared to women who had a normal weight. In the 1946–51 cohort being a current smoker and reporting difficulty managing on their income also increased the odds of diabetes while having a university level of education was associated with a decrease in odds of diabetes. The relatively higher diabetes prevalence and stronger association with obesity in this cohort of women mirror the fact that these women had both the highest baseline BMI and also weight gain over time compared to all other three cohorts of ALSWH [41].

These increasing trends in diabetes prevalence with age are in line with other studies [35, 42, 43]. For instance, a longitudinal study of diabetes prevalence in the US–based on survey and measurement of glycated haemoglobin levels for undiagnosed diabetes–reported a 72% overall increase in prevalence between 1999 and 2016, with 40% of this increase attributed to change in BMI and population aging [44]. In these data, diabetes prevalence rose with age within each birth cohort, and also increased between the birth cohorts [45]. Similarly, a study from Mexico shows large cohort increases in the prevalence of diabetes, likely due to changes in nutrition and physical activity [46]. An earlier study in Australia found increasing trends in age-standardized prevalence of diabetes from 2002 (6.2%) to 2013 (7.9%), with strong cohort effects that mirror cohort patterns for obesity. However, the study did not discuss if correction for differences in overweight/obesity prevalence changes the pattern of diabetes prevalence across cohorts [47]. Other explanations for the increasing diabetes prevalence over time, found here and in other studies, including improved diabetes diagnosis techniques, better disease management, longer patient survival, population aging, and or a real increase in diabetes incidence.

The increasing prevalence of diabetes in younger cohorts sends an important message to healthcare practitioners and policymakers responsible for the planning of disease prevention and management interventions. There are multiple avenues to prevent diabetes in younger cohorts, particularly related to achievement and maintenance of normal weight and engagement in physical exercise. Such lifestyle modifications were associated with up to 58% reduction in the incidence of type 2 diabetes among people with impaired glucose tolerance, 40–50% of whom would normally progress to diabetes [48, 49]. Similarly, pharmacological interventions were effective in the prevention of diabetes in people with impaired glucose tolerance [49, 50].

### Strengths and limitations

The use of multiple data sources to ascertain diabetes cases is a major strength of this analysis, and enhanced the completeness of case ascertainment. The selection of two large-sized population-representative cohorts of women was another strength of the study. The prospective study design may also have reduced recall bias in the surveys. An important limitation of this study is related to the assumptions made to construct the diabetes case identifying algorithms. For instance, if a woman had any diabetes indicator in her hospital admission records, she was considered as having diabetes without any further investigation. However, our approach should result in more validity and complete case ascertainment compared to other studies, which used a single method to identify people with diabetes. Another limitation of this study is the possibility for some women with diabetes to be classified as false negative or as having uncertain diabetes status due to the stringent case ascertainment algorithms we employed. However, the women who were classified as having uncertain diabetes status appeared to be dissimilar from both of the other groups i.e. the women with and without diabetes. Despite this, we believe this would be one of the most reliable non-biomarker based methods of diabetes case ascertainment and prevalence estimation.

## Conclusions

This study demonstrated the importance of using multiple data sources to improve the completeness of diabetes case ascertainment. The findings of this study imply that there is a large potential for health care administrative data sources to be used in diabetes and other chronic disease ascertainment and precise prevalence estimation. This may have a huge practical importance in improving the precision of disease burden and health care need estimation. The study found that diabetes prevalence has been continuously increasing with age in both cohorts of women during the period 1996 to 2016. However, higher prevalence rates were observed among the 1946–51 cohort women at a much earlier age compared to the 1921–26 cohort. Having diabetes was significantly associated with lifestyle factors such as physical activity and obesity. A stronger association was observed between being obese and having diabetes in the 1946–51 cohort. The findings imply that the prevalence of diabetes will continue to increase in future cohorts of women. This emphasizes the need for interventions aimed at diabetes prevention, early identification, and improved management. Importantly, addressing overweight and obesity management and increasing physical activity should be part of diabetes prevention efforts.

## Supporting information

S1 Table Medicare benefits schedule health services included in diabetes case identifying algorithms. (DOCX)

S2 Table ATC-5 codes for diabetes medications used in the identification of patients with diabetes from the Pharmaceutical Benefits Scheme dataset. (DOCX)

S3 Table Number and percentage of the total number of women with diabetes identified from each of the four data sources in the 1921–26 and 1946–51 cohort women. (DOCX)

S4 Table Comparison of baseline characteristics of women with diabetes, without diabetes and uncertain diabetes status in the 1921–1926 and 1946–51 cohort. (DOCX)

## References

[1] Mendis S, Alwan A, eds. Prioritized research agenda for prevention and control of non-communicable diseases. Geneva, World Health Organization 2011.

[2] Ramke J, Lee L, Brian G. Prevalence of diabetes among adults aged >/ = 40 years in Timor-Leste. Journal of diabetes. 2012;4(4):392–4. 10.1111/j.1753-0407.2012.00217.x 22727126

[3] WHO. Global report on diabetes 2016 [cited 2017 July 12]. http://apps.who.int/iris/bitstream/10665/204871/1/9789241565257_eng.pdf].

[4] Cho NH, Shaw JE, Karuranga S, Huang Y, da Rocha Fernandes JD, Ohlrogge AW, et al IDF Diabetes Atlas: Global estimates of diabetes prevalence for 2017 and projections for 2045. Diabetes research and clinical practice. 2018;138:271–81. 10.1016/j.diabres.2018.02.023 29496507

[5] International Diabetes Federation. Advocacy guide to the IDF Diabetes Atlas Ninth edition 2019. [cited 2019 November 18]. https://diabetesatlas.org/upload/resources/2019/2019_IDF_Advocacy_guide.pdf.

[6] Deshpande AD, Harris-Hayes M, Schootman M. Epidemiology of diabetes and diabetes-related complications. Physical therapy. 2008;88(11):1254–64. 10.2522/ptj.20080020 18801858 PMC3870323

[7] Classification of diabetes mellitus. Geneva: World Health Organization; 2019. Licence: CC BY-NC-SA 3.0 IGO.

[8] Brown W, Burton N, Heesch K. Physical activity and health in mid age and older women. Canberra: The Office for Women, Department of Families, Community Services, and Indigenous Affairs. 2007. Report No.: 978-1-921380-77-8.

[9] Bannier K, Lichtenauer M, Franz M, Fritzenwanger M, Kabisch B, Figulla HR, et al Impact of diabetes mellitus and its complications: survival and quality-of-life in critically ill patients. Journal of diabetes and its complications. 2015;29(8):1130–5. 10.1016/j.jdiacomp.2015.08.010 26361811

[10] Cheng S-W, Wang C-Y, Chen J-H, Ko Y. Healthcare costs and utilization of diabetes-related complications in Taiwan: A claims database analysis. Medicine. 2018;97(31):e11602–e. 10.1097/MD.0000000000011602 30075532 PMC6081128

[11] Lin S, Tukana I, Linhart C, Morrell S, Taylor R, Vatucawaqa P, et al Diabetes and obesity trends in Fiji over 30 years. Journal of diabetes. 2016;8(4):533–43. 10.1111/1753-0407.12326 26201444

[12] Australian Institute of Health and Welfare. Australia’s health 2016 [cited 2018 July 19]. http://www.aihw.gov.au/WorkArea/DownloadAsset.aspx?id=60129555788.

[13] Van Netten JJ, Lazzarini PA, Fitridge R, Kinnear E, Griffiths I, Malone M, et al Australian diabetes-related foot disease strategy 2018–2022: The first step towards ending avoidable amputations within a generation. Brisbane: Diabetic Foot Australia, Wound Management CRC; 2017 2017.

[14] Papoz L, Balkau B, Lellouch J. Case counting in epidemiology: limitations of methods based on multiple data sources. International journal of epidemiology. 1996;25(3):474–8. 10.1093/ije/25.3.474 8671546

[15] Kleefstra N, Landman GW, Van Hateren KJ, Meulepas M, Romeijnders A, Rutten GE, et al Dutch diabetes prevalence estimates (DUDE-1). Journal of diabetes. 2016;8(6):863–5. 10.1111/1753-0407.12370 26694523

[16] Taylor R, Zimmet P, Naseri T, Hufanga S, Tukana I, Magliano DJ, et al Erroneous inflation of diabetes prevalence: Are there global implications? Journal of diabetes. 2016;8(6):766–9. 10.1111/1753-0407.12447 27400903

[17] Morgan CL, Currie CJ, Stott NC, Smithers M, Butler CC, Peters JR. Estimating the prevalence of diagnosed diabetes in a health district of Wales: the importance of using primary and secondary care sources of ascertainment with adjustment for death and migration. Diabetic medicine: a journal of the British Diabetic Association. 2000;17(2):141–5. 10746485 10.1046/j.1464-5491.2000.00221.x

[18] Althubaiti A. Information bias in health research: definition, pitfalls, and adjustment methods. J Multidiscip Healthc. 2016;9:211–7. 10.2147/JMDH.S104807 27217764 PMC4862344

[19] Webbie K, O’Brien K. Use of medicines by Australians with diabetes. Bulletin no. 47. AIHW cat. no. AUS 82. Canberra: AIHW.; 2006.

[20] Amed S, Dean HJ, Panagiotopoulos C, Sellers EA, Hadjiyannakis S, Laubscher TA, et al Type 2 diabetes, medication-induced diabetes, and monogenic diabetes in Canadian children: a prospective national surveillance study. Diabetes Care. 2010;33(4):786–91. 10.2337/dc09-1013 20067956 PMC2845028

[21] Anwar H, Fischbacher CM, Leese GP, Lindsay RS, McKnight JA, Wild SH. Assessment of the under-reporting of diabetes in hospital admission data: a study from the Scottish Diabetes Research Network Epidemiology Group. Diabetic medicine: a journal of the British Diabetic Association. 2011;28(12):1514–9. 21883441 10.1111/j.1464-5491.2011.03432.x PMC4215191

[22] Haut ER, Pronovost PJ, Schneider EB. Limitations of administrative databases. Jama. 2012;307(24):2589; author reply -90. 10.1001/jama.2012.6626 22735421

[23] Harvey JN, Craney L, Kelly D. Estimation of the prevalence of diagnosed diabetes from primary care and secondary care source data: comparison of record linkage with capture-recapture analysis. J Epidemiol Community Health. 2002;56(1):18–23. 10.1136/jech.56.1.18 11801615 PMC1731996

[24] Waller M, Mishra GD, Dobson AJ. Estimating the prevalence of dementia using multiple linked administrative health records and capture-recapture methodology. Emerg Themes Epidemiol. 2017;14:3-. 10.1186/s12982-017-0057-3 28261312 PMC5327574

[25] Dobson AJ, Hockey R, Brown WJ, Byles JE, Loxton DJ, McLaughlin D, et al Cohort Profile Update: Australian Longitudinal Study on Women’s Health. International journal of epidemiology. 2015;44(5):1547,a-f 10.1093/ije/dyv110 26130741

[26] Lee C, Dobson AJ, Brown WJ, Bryson L, Byles J, Warner-Smith P, et al Cohort Profile: the Australian Longitudinal Study on Women’s Health. International journal of epidemiology. 2005;34(5):987–91. 10.1093/ije/dyi098 15894591

[27] Duckett SJ, Willcox S. The Australian health care system Fifth edition ed. South Melbourne, Victoria, Australia: Oxford University Press; 2015 2015. 448 p.

[28] Knott RJ, Clarke PM, Heeley EL, Chalmers JP. Measuring the Progressivity of the Pharmaceutical Benefits Scheme. The Australian Economic Review. 2015;48(2):122–32.

[29] Centre for Health Record Linkage. ICD-10-AM/ACHI/ACS [cited 2017 July 11]. https://ace.ihpa.gov.au/Icd10.aspx.

[30] Australian Institute of Health and Welfare. National Death Index (NDI) (AIHW) 2017 [http://www.aihw.gov.au/national-death-index/.

[31] Bullard KM, Cowie CC, Lessem SE, Saydah SH, Menke A, Geiss LS, et al Prevalence of Diagnosed Diabetes in Adults by Diabetes Type—United States, 2016. MMWR Morb Mortal Wkly Rep. 2018;67(12):359–61. 10.15585/mmwr.mm6712a2 29596402 PMC5877361

[32] Joshy G, Porter T, Le Lievre C, Lane J, Williams M, Lawrenson R. Prevalence of diabetes in New Zealand general practice: the influence of ethnicity and social deprivation. J Epidemiol Community Health. 2009;63(5):386–90. 10.1136/jech.2008.078873 19211590

[33] Leikauf J, Federman AD. Comparisons of self-reported and chart-identified chronic diseases in inner-city seniors. Journal of the American Geriatrics Society. 2009;57(7):1219–25. 10.1111/j.1532-5415.2009.02313.x 19486197 PMC2768322

[34] Okura Y, Urban LH, Mahoney DW, Jacobsen SJ, Rodeheffer RJ. Agreement between self-report questionnaires and medical record data was substantial for diabetes, hypertension, myocardial infarction and stroke but not for heart failure. Journal of clinical epidemiology. 2004;57(10):1096–103. 10.1016/j.jclinepi.2004.04.005 15528061

[35] NCD Risk Factor Collaboration. Worldwide trends in diabetes since 1980: a pooled analysis of 751 population-based studies with 4·4 million participants. The Lancet. 2016;387(10027):1513–30. 10.1016/S0140-6736(16)00618-8 PMC5081106 27061677

[36] Akushevich I, Yashkin AP, Kravchenko J, Fang F, Arbeev K, Sloan F, et al Identifying the causes of the changes in the prevalence patterns of diabetes in older U.S. adults: A new trend partitioning approach. Journal of diabetes and its complications. 2018;32(4):362–7. 10.1016/j.jdiacomp.2017.12.014 29433960 PMC5849520

[37] Bloomgarden Z, Ning G. Diabetes and aging. Journal of diabetes. 2013;5(4):369–71. 10.1111/1753-0407.12086 24004480

[38] Byles J, Hockey R, McLaughlin D, Dobson A, Brown W, Loxton DJ, et al. Chronic conditions, physical function and health care use: Findings from the Australian Longitudinal Study on Women’s Health. Report prepared for the Australian Government Department of Health, June 2015.; 2015.

[39] Andersson T, Ahlbom A, Magnusson C, Carlsson S. Prevalence and incidence of diabetes in Stockholm County 1990–2010. PloS one. 2014;9(8):e104033–e. 10.1371/journal.pone.0104033 25121976 PMC4133405

[40] Iwasa T, Amiya E, Ando J, Watanabe M, Murasawa T, Komuro I. Different Contributions of Physical Activity on Arterial Stiffness between Diabetics and Non-Diabetics. PloS one. 2016;11(8):e0160632 10.1371/journal.pone.0160632 27508936 PMC4980026

[41] Mishra G, Chan H, Hockey R, Waller M, Kanesarajah J, Byles J, et al. Future health service use and cost: Insights from the Australian Longitudinal Study on Women’s Health. Report prepared for the Australian Government Department of Health, June 2016.; 2016.

[42] Gonzalez EL, Johansson S, Wallander MA, Rodriguez LA. Trends in the prevalence and incidence of diabetes in the UK: 1996–2005. J Epidemiol Community Health. 2009;63(4):332–6. 10.1136/jech.2008.080382 19240084

[43] Huang X-b, Tang W-w, Liu Y, Hu R, Ouyang L-y, Liu J-x, et al Prevalence of diabetes and unrecognized diabetes in hypertensive patients aged 40 to 79 years in southwest China. PloS one. 2017;12(2):e0170250 10.1371/journal.pone.0170250 28192474 PMC5305248

[44] Fang M. Trends in the Prevalence of Diabetes Among U.S. Adults: 1999–2016. Am J Prev Med. 2018;55(4):497–505. 10.1016/j.amepre.2018.05.018 30126668

[45] Fishman EI, Stokes A, Preston SH. The Dynamics of Diabetes among Birth Cohorts in the United States. Diabetes care. 2014:DC_131982. 10.2337/dc13-1982 PMC3964490 24513590

[46] Meza R, Barrientos-Gutierrez T, Rojas-Martinez R, Reynoso-Noveron N, Palacio-Mejia LS, Lazcano-Ponce E, et al Burden of type 2 diabetes in Mexico: past, current and future prevalence and incidence rates. Preventive medicine. 2015;81:445–50. 10.1016/j.ypmed.2015.10.015 26546108 PMC4679631

[47] Taylor AW, Shi Z, Montgomerie A, Dal Grande E, Campostrini S. The use of a chronic disease and risk factor surveillance system to determine the age, period and cohort effects on the prevalence of obesity and diabetes in South Australian adults—2003-2013. PloS one. 2015;10(4):e0125233 10.1371/journal.pone.0125233 25923664 PMC4414468

[48] Lindstrom J, Peltonen M, Eriksson JG, Ilanne-Parikka P, Aunola S, Keinanen-Kiukaanniemi S, et al Improved lifestyle and decreased diabetes risk over 13 years: long-term follow-up of the randomised Finnish Diabetes Prevention Study (DPS). Diabetologia. 2013;56(2):284–93. 10.1007/s00125-012-2752-5 23093136

[49] DeFronzo RA, Abdul-Ghani M. Type 2 diabetes can be prevented with early pharmacological intervention. Diabetes Care. 2011;34 Suppl 2:S202–9. 21525456 10.2337/dc11-s221 PMC3632162

[50] Gillies CL, Abrams KR, Lambert PC, Cooper NJ, Sutton AJ, Hsu RT, et al Pharmacological and lifestyle interventions to prevent or delay type 2 diabetes in people with impaired glucose tolerance: systematic review and meta-analysis. BMJ (Clinical research ed). 2007;334(7588):299-. 10.1136/bmj.39063.689375.55 PMC1796695 17237299

