# Supplementary material for: Age and cohort rise in diabetes prevalence among older Australian women: Case ascertainment using survey and healthcare administrative data
Source: PLoS One. 2020 Jun 18;15(6):e0234812. doi: 10.1371/journal.pone.0234812 (PMC7302694; doi:10.1371/journal.pone.0234812)
Supplement: S1 Table — (DOCX) [file pone.0234812.s001.docx]

Table S1: Medicare Benefits Schedule health services included in diabetes case identifying algorithms

| Types of diabetes service | MBS item numbers* |
| --- | --- |
| 1. Group allied health services for people with type 2 diabetes | 81100, 81105, 81110, 81115, 81120, 81125 |
| 1. Annual diabetes cycle of care completion for patients with established diabetes mellitus | 2517, 2518, 2521, 2522, 2525, 2526, 2620, 2622, 2624 |
| 1. Pathology tests and other diabetes services |  |
| - 1. HbA1c test (for management of established diabetes) | 66551 |
| - 1. HbA1c test for monitoring purposes, Aboriginal Medical Service (for management of established diabetes) | 73840 |
| - 1. Quantitation of fructosamine performed in the management of established diabetes | 66557 |
| - 1. Diabetes health education service | 10951 |
| - 1. Diabetes health education service provided to a person who is of Aboriginal or Torres Strait Islander descent | 81305 |
| - 1. Professional attendance of more than 15 minutes duration, being the first in a course of attention involving the examination of the eyes, with the instillation of a mydriatic, of a patient with diabetes mellitus requiring comprehensive reassessment | 10915 |

*Item numbers with no recorded use during the period 01 January 1996 to 31 December 2015 were excluded. Microalbumin quantitation in urine (66560) is also excluded due to its lack of specificity and most of the true positives from this item number can be captured by other item numbers.
