# Supplementary material for: Age and cohort rise in diabetes prevalence among older Australian women: Case ascertainment using survey and healthcare administrative data
Source: PLoS One. 2020 Jun 18;15(6):e0234812. doi: 10.1371/journal.pone.0234812 (PMC7302694; doi:10.1371/journal.pone.0234812)
Supplement: S2 Table — (DOCX) [file pone.0234812.s002.docx]

Table S2: ATC-5 codes for diabetes medications used in identification of patients with diabetes from the Pharmaceutical Benefits Scheme dataset

| Pharmacological classes of drugs | Drugs | ATC-5 codes |
| --- | --- | --- |
| Blood glucose lowering drugs, excluding insulins |  |  |
| Biguanides | Metformin | A10BA02 |
| Sulfonylureas | Glyburide or Glibenclamide | A10BB01 |
|  | Gliclazide | A10BB09 |
|  | Glimepiride | A10BB12 |
|  | Glipizide | A10BB07 |
| Combinations of oral blood glucose lowering drugs | Metformin + Glibenclamide | A10BD02 |
|  | Metformin + Rosiglitazone | A10BD03 |
|  | Metformin + Sitagliptin | A10BD07 |
|  | Metformin + Vildagliptin | A10BD08 |
|  | Metformin + Saxagliptin | A10BD10 |
|  | Metformin + Linagliptin | A10BD11 |
|  | Metformin + Alogliptin | A10BD13 |
|  | Metformin + Dapagliflozin | A10BD15 |
| Alpha glucosidase inhibitors | Acarbose | A10BF01 |
| Thiazolidinediones | Pioglitazone | A10BG03 |
|  | Rosiglitazone | A10BG02 |
| Dipeptidyl peptidase 4 (DPP-4) inhibitors | Linagliptin | A10BH05 |
|  | Saxagliptin | A10BH03 |
|  | Sitagliptin | A10BH01 |
|  | Vildagliptin | A10BH02 |
|  | Alogliptin | A10BH04 |
|  | [Sitagliptin + Simvastatin](https://www.whocc.no/atc_ddd_index/?code=A10BH51&showdescription=yes) | A10BH51 |
| Sodium-glucose co-transporter 2 (SGLT-2) inhibitors | [Dapagliflozin](https://www.whocc.no/atc_ddd_index/?code=A10BK01&showdescription=yes) | A10BK01 |
|  | [Canagliflozin](https://www.whocc.no/atc_ddd_index/?code=A10BK02&showdescription=yes) | A10BK02 |
|  | Empagliflozin | A10BK03 |
|  | [Ertugliflozin](https://www.whocc.no/atc_ddd_index/?code=A10BK04&showdescription=yes) | A10BK04 |
| Other blood glucose lowering drugs, excluding insulins | Exenatide | A10BX04 |
| Insulin and insulin analogues | Insulin lispro | A10AB04 |
|  | Insulin aspart | A10AB05 |
|  | Insulin aspart + Insulin aspart Protamine | A10AD |
|  | Insulin Neutral Human | A10AB01 |
|  | Insulin Neutral Bovine | A10AB02 |
|  | Insulin Glulisine | A10AB06 |
|  | Insulin protaphane | A10AC01 |
|  | Insulin Isophane Bovine | A10AC02 |
|  | Insulin Isophane Human + Insulin Neutral Human | A10AD01 |
|  | Insulin Lispro + Insulin Lispro Protamine | A10AD04 |
|  | Insulin Zinc Suspension (Crystalline)(Ultralente) | A10AE01 |
|  | Insulin glargine | A10AE04 |
|  | Insulin detemir | A10AE05 |
