# Supplementary material for: Age and cohort rise in diabetes prevalence among older Australian women: Case ascertainment using survey and healthcare administrative data
Source: PLoS One. 2020 Jun 18;15(6):e0234812. doi: 10.1371/journal.pone.0234812 (PMC7302694; doi:10.1371/journal.pone.0234812)
Supplement: S3 Table — (DOCX) [file pone.0234812.s003.docx]

Table S3 – Number and percentage of the total number of women with diabetes identified from each of the four data sources in the 1921-26 and 1946-51 cohort women

| Sources | 1921-26 cohort | 1946-51 cohort |
| --- | --- | --- |
| ALSWH | 1827 (68.5) | 1430 (70.2) |
| MBS | 2123 (79.6) | 1762 (86.5) |
| PBS | 1474 (55.3) | 1339 (65.7) |
| APDC | 1255 (47.1) | 664 (32.6) |
| Total | 2667 | 2037 |
