# Supplementary material for: Age and cohort rise in diabetes prevalence among older Australian women: Case ascertainment using survey and healthcare administrative data
Source: PLoS One. 2020 Jun 18;15(6):e0234812. doi: 10.1371/journal.pone.0234812 (PMC7302694; doi:10.1371/journal.pone.0234812)
Supplement: S4 Table — (DOCX) [file pone.0234812.s004.docx]

Table S4–Comparison of baseline characteristics of women with diabetes, without diabetes and uncertain diabetes status in the 1921-1926 and 1946-51 cohort

|  |  | 1921-26 cohort | | | |  | 1946-51 cohort | | | |
| --- | --- | --- | --- | --- | --- | --- | --- | --- | --- | --- |
| Predictors | Diabetes (n=2667) | | No diabetes (n=8875) | Uncertain diabetes (n=890) | p | Diabetes (n=2037) | | No diabetes (n=10650) | Uncertain diabetes (n=1027) | p |
| Highest level of education |  | |  |  | <0.0001 |  | |  |  | <0.0001 |
| Year 12 or below | 2216 (88.1) | | 7073 (84.2) | 737 (86.3) |  | 1516 (75.7) | | 6876 (65.2) | 693 (67.9) |  |
| Trade certificate/diploma | 224 (8.9) | | 998 (11.9) | 89 (10.4) |  | 303 (15.1) | | 2100 (19.9) | 196 (19.2) |  |
| University degree | 75 (3.0) | | 328 (3.9) | 28 (3.3) |  | 184 (9.2) | | 1577 (14.9) | 131 (12.8) |  |
| Area of residence |  | |  |  | 0.10 |  | |  |  | 0.57 |
| Major cities | 1090 (40.9) | | 3675 (41.4) | 408 (45.8) |  | 721 (35.4) | | 3879 (36.4) | 399 (38.9) |  |
| Inner regional | 1012 (38.0) | | 3479 (39.2) | 312 (35.1) |  | 778 (38.2) | | 4094 (38.4) | 342 (33.3) |  |
| Outer regional/remote/very remote | 565 (21.2) | | 1721 (19.4) | 170 (19.1) |  | 538 (26.4) | | 2676 (25.1) | 286 (27.9) |  |
| Marital status |  | |  |  | 0.03 |  | |  |  | 0.12 |
| Partnered^*^ | 1444 (54.4) | | 5066 (57.2) | 510 (57.6) |  | 1650 (81.4) | | 8805 (83.1) | 856 (83.8) |  |
| Not partnered | 1212 (45.6) | | 3786 (42.8) | 375 (42.4) |  | 378 (18.6) | | 1791 (16.9) | 166 (16.2) |  |
| BMI classification |  | |  |  | <0.0001 |  | |  |  | <0.0001 |
| Normal weight (18.5≤BMI<25) | 902 (35.4) | | 4618 (54.0) | 425 (49.8) |  | 413 (21.6) | | 5820 (56.6) | 432 (44.1) |  |
| Underweight (BMI<18.5) | 35 (1.4) | | 339 (4.0) | 17 (2.0) |  | 11 (0.6) | | 207 (2.0) | 17 (1.7) |  |
| Overweight (25≤BMI<30) | 979 (38.4) | | 2684 (31.4) | 297 (34.8) |  | 615 (32.1) | | 2888 (28.1) | 311 (31.7) |  |
| Obese (BMI≥30) | 634 (24.9) | | 913 (10.7) | 115 (13.5) |  | 877 (45.8) | | 1377 (13.4) | 220 (22.5) |  |
| Level of physical activity |  | |  |  | <0.0001 |  | |  |  | <0.0001 |
| Moderate | 305 (14.3) | | 1136 (16.0) | 137 (19.4) |  | 338 (21.3) | | 1961 (22.3) | 184 (21.6) |  |
| Nil/sedentary | 870 (40.7) | | 2294 (32.2) | 198 (28.0) |  | 383 (24.1) | | 1493 (17.0) | 152 (17.9) |  |
| Low | 609 (28.5) | | 2144 (30.1) | 609 (28.5) |  | 485 (30.6) | | 2707 (30.8) | 278 (32.7) |  |
| High | 353 (16.5) | | 1546 (21.7) | 131 (18.5) |  | 381 (24.0) | | 2627 (29.9) | 237 (27.9) |  |
| Smoking status |  | |  |  | 0.0001 |  | |  |  | 0.0001 |
| Never smoked | 1604 (64.8) | | 5134 (61.8) | 505 (61.2) |  | 982 (50.1) | | 5555 (53.8) | 512 (51.9) |  |
| Ex-smoker | 728 (29.4) | | 2470 (29.7) | 260 (31.5) |  | 547 (27.9) | | 2946 (28.6) | 280 (28.4) |  |
| Current smoker | 142 (5.7) | | 710 (8.5) | 60 (7.3) |  | 430 (22.0) | | 1817 (17.6) | 195 (19.8) |  |
| Difficulty to manage on income |  | |  |  | <0.0001 |  | |  |  | <0.0001 |
| Easy | 1883 (71.1) | | 6578 (74.5) | 611 (69.2) |  | 946 (46.7) | | 6175 (58.3) | 556 (54.5) |  |
| Difficult | 767 (28.9) | | 2251 (25.5) | 272 (30.8) |  | 1078 (53.3) | | 4409 (41.7) | 464 (45.5) |  |
| Private hospital insurance |  | |  |  | <0.0001 |  | |  |  | <0.0001 |
| No | 4754 (54.0) | | 1582 (59.8) | 452 (51.3) |  | 1178 (58.0) | | 5473 (51.5) | 515 (50.2) |  |
| Yes | 4054 (46.0) | | 1062 (40.2) | 430 (48.8) |  | 854 (42.0) | | 5165 (48.6) | 511 (49.8) |  |

*Married or in a de facto relationship, BMI: Body mass index
